# A Comprehensive Framework for the Development of a Compact, Cost-Effective, and Robust Hyperspectral Camera Using COTS Components and a VPH Grism

**DOI:** 10.3390/s25123631

**Published:** 2025-06-10

**Authors:** Sukrit Thongrom, Panuwat Pengphorm, Surachet Wongarrayapanich, Apirat Prasit, Chanisa Kanjanasakul, Wiphu Rujopakarn, Saran Poshyachinda, Chalongrat Daengngam, Nawapong Unsuree

**Affiliations:** 1 Division of Physical Science, Faculty of Science, Prince of Songkla University, Hat Yai District, Songkhla 90110, Thailand; sukrit@narit.or.th (S.T.); panuwat@narit.or.th (P.P.); chalongrat.d@psu.ac.th (C.D.); 2 National Astronomical Research Institute of Thailand (Public Organization), Mae Rim District, Chiang Mai 50180, Thailand; surachet@narit.or.th (S.W.); apirat@narit.or.th (A.P.); chanisa@narit.or.th (C.K.); wiphu@narit.or.th (W.R.); saran@narit.or.th (S.P.); 3 Department of Physics, Academic Division, Chulachomklao Royal Military Academy, Muang, Nakhon Nayok 26001, Thailand

**Keywords:** hyperspectral imaging (HSI), grism-based hyperspectral camera, commercial off-the-shelf (COTS) components, volume phase holographic (VPH) grism, on-axis optical layout

## Abstract

Hyperspectral imaging (HSI) is an effective technique for material identification and classification, utilizing spectral signatures with applications in remote sensing, environmental monitoring, and allied disciplines. Despite its potential, the broader adoption of HSI technology is hindered by challenges related to compactness, affordability, and durability, exacerbated by the absence of standardized protocols for developing practical hyperspectral cameras. This study introduces a comprehensive framework for developing a compact, cost-effective, and robust hyperspectral camera, employing commercial off-the-shelf (COTS) components and a volume phase holographic (VPH) grism. The use of COTS components reduces development time and manufacturing costs while maintaining adequate performance, thereby improving accessibility for researchers and engineers. The incorporation of a VPH grism enables an on-axis optical design, enhancing compactness, reducing alignment sensitivity, and improving system robustness. The proposed framework encompasses spectrograph design, including optical simulations and tolerance analysis conducted in ZEMAX OpticStudio, alongside assembly procedures, performance assessment, and hyperspectral image acquisition via a pushbroom scanning approach, all integrated into a structured, step-by-step workflow. The resulting prototype, housed in an aluminum enclosure, operates within the 420–830 nm wavelength range, achieving a spectral resolution of 2 nm across 205 spectral bands. It effectively differentiates vegetation, water, and built structures, resolves atmospheric absorption features, and demonstrates the ability to distinguish materials in low-light conditions, providing a scalable and practical advancement in HSI technology.

## 1. Introduction

Hyperspectral imaging (HSI) is a powerful technique that captures detailed spectral data for each pixel, allowing precise identification and classification of materials based on their unique spectral signatures [1]. By acquiring hundreds of continuous spectral bands across the electromagnetic spectrum, HSI detects subtle differences in composition, structure, and condition that traditional RGB or multispectral imaging methods often miss. This capability makes HSI especially useful in fields such as environmental monitoring [2,3,4], precision agriculture [5,6,7], biomedical diagnostics [8,9], food safety inspection [10,11], and defense [12,13]. However, the practical use of hyperspectral cameras requires careful optimization of compactness, cost-efficiency, and robustness to ensure broad adoption and reliable performance across diverse operational environments and platforms.

The increasing demand for compact, cost-effective, and robust hyperspectral imaging systems has spurred research into innovative optical configurations. Compactness is vital for applications such as unmanned aerial vehicles (UAVs), satellites, and other portable platforms, where size and weight are key limitations. Cost-effectiveness is also a crucial factor in promoting the widespread adoption of hyperspectral imaging technology. The use of commercial off-the-shelf (COTS) components provides a practical way to lower manufacturing costs and shorten development cycles while ensuring acceptable performance levels [14,15]. In addition, system robustness is a key requirement for ensuring the long-term reliability of hyperspectral imaging systems in harsh operational environments. These systems must endure mechanical vibrations, temperature fluctuations, and other environmental stressors while maintaining acceptable optical performance. Most HSI cameras use an off-axis optical layout, such as the Lens-Grating-Lens design [14,16], the Czerny-Turner configuration [17], or the Offner layout [18]. However, the off-axis design complicates the alignment process due to variations in off-axis angles, requiring precise alignment of each optical component. As a result, their optical performance is often vulnerable to misalignments caused by vibrations or assembly errors. For example, Xue et al. (2021) reported that their UAV-mounted Offner-based spectrometer, while providing excellent spectral fidelity, demanded extensive structural modeling and vibration analysis to maintain stability during flight [18]. Moreover, the folded and decentered optical paths typical of off-axis geometries usually lead to larger system volumes and increased design complexity, posing challenges for miniaturization and modular integration into compact platforms. These drawbacks have sparked growing interest in on-axis architecture, which provides benefits such as improved mechanical robustness, simpler alignment procedures, and reduced instrument size. One effective approach employs a volume phase holographic (VPH) grism, a hybrid optical element that integrates a VPH grating with a prism, also referred to as a prism-grating-prism (PGP) configuration. This design enables an on-axis optical layout, eliminating off-axis angles and reducing alignment difficulties, thereby enhancing system robustness. Additionally, compared to traditional diffraction gratings, VPH grisms deliver higher diffraction efficiency across a broad wavelength range while minimizing stray light and ghosting [19,20]. This improves spectral purity and enhances the overall accuracy and reliability of hyperspectral imaging systems.

Grism-based hyperspectral cameras have been applied to advanced analysis in various fields, including mineral exploration, biomedical imaging, cultural heritage preservation, and precision agriculture. For example, Wu et al. (2014) [21] developed a lightweight airborne prism-grating-prism (PGP) hyperspectral system for remote sensing in mineral exploration, featuring two PGP spectrographs: one for visible-to-near-infrared (VNIR) and another for short-wave infrared (SWIR). This system was tested in airborne field experiments. Zhu et al. (2024) [22] introduced a dual-mode microscopic hyperspectral imaging system, operating in transmission and fluorescence modes, integrated with hybrid deep neural networks (DNNs) for simultaneous detection of multiple pathogenic bacteria, improving clinical diagnostic accuracy. Jiao et al. (2021) [23] utilized a similar dual-mode system, combining reflection and fluorescence modes with machine learning algorithms, to classify varieties of *Tetrastigma hemsleyanum*, a medicinal plant. Zhang et al. (2016) [24] demonstrated the effectiveness of a portable VNIR hyperspectral imaging system, equipped with a commercial PGP spectrograph from Specim, in distinguishing sun-dried from sulfur-fumigated Chinese herbs, highlighting its value in quality control for herbal medicine. Similarly, Dirk et al. (2009) [25] used a VNIR hyperspectral imaging system with a PGP spectrograph from Specim to analyze pigments and artwork deterioration in Old Master paintings and 19th-century watercolors, offering key insights for cultural heritage preservation. In precision agriculture, Krtalić et al. (2019) [26] employed a VNIR hyperspectral imaging system with a commercial ImSpector V9 PGP spectrograph (spectral range: 430 to 500 nm, spectral resolution: 4.4 nm) to survey vineyards and calculate vegetation indices, proving its utility in crop monitoring. Likewise, Pengphorm et al. (2024) [5] used a VNIR hyperspectral imaging system with a grism-based spectrograph from Shelyak Instruments to measure leaf chlorophyll content (LCC) in rice leaves, emphasizing its potential for agricultural applications.

Despite its application across these diverse fields, standardized, step-by-step development protocols for on-axis grism-based HSI cameras remain absent, potentially restricting broader adoption and innovation in the field. To address this gap, this paper presents a comprehensive framework for developing on-axis HSI cameras using COTS components and a VPH grism. The framework includes spectrograph design with optical simulations and tolerance analysis, assembly procedures, performance evaluation, and hyperspectral image acquisition via a pushbroom scanning method, all integrated into a cohesive and structured development process. Our prototype operates within a VNIR spectral range of 420 to 830 nm, achieving a spectral resolution of 2 nm across 205 spectral bands. It effectively distinguishes vegetation, water, and built structures while also detecting atmospheric absorption features. It also demonstrates the ability to differentiate materials in low-light environments. By offering this detailed methodology as a practical guideline, we aim to promote the widespread adoption of compact, cost-effective, and robust hyperspectral imaging systems across various scientific and industrial applications. In brief, this study’s primary contributions are summarized as follows: (1) providing a comprehensive guideline for developing HSI systems, (2) facilitating broader research adoption by enabling the construction of cost-effective HSI cameras with high optical performance in the VNIR regime, and (3) highlighting the benefits of a VPH grism, which simplifies the assembly and integration process and enhances the compactness and robustness of HSI systems.

## 2. Related Work

The proposed HSI system utilizes an on-axis configuration facilitated by a VPH grism. This design significantly simplifies the alignment and integration process and enhances the compactness and robustness of the system. Table 1 compares the optical performance in a VNIR spectral range of our HSI system with other on-axis HSI systems including ImSpector V8E, V9, and N10E models from Specim (Druzik et al. [25]; Krtalić et al. [26]; Cucci et al. [27]). The optical performance of our system is comparable to that of commercial HSI systems. However, existing systems lack a detailed, step-by-step construction workflow. To address this gap, our paper presents a comprehensive and systematic framework that covers both theoretical and practical aspects, enabling researchers to develop compact, cost-effective, and robust on-axis HSI cameras.

## 3. Development Process

**Figure 1:**
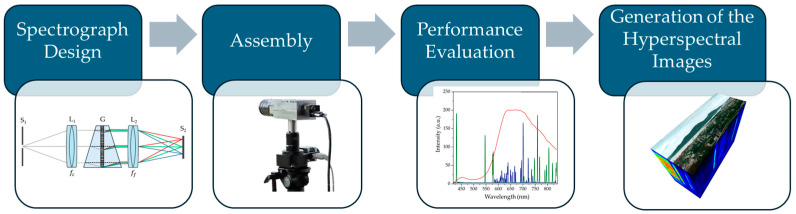
Development process of HSI system.

The development of the HSI system, as shown in Figure 1, involves several key stages: spectrograph design, assembly, performance evaluation, and hyperspectral image generation. The optical performance of the spectrograph, including spectral coverage and resolution, was simulated using ZEMAX OpticStudio (version 23.2.1) and Python (version 3.11.5). This analysis determined critical design parameters, such as the focal lengths of the collimating and focusing lenses, the groove density of the diffraction grating, and the dimensions and separation distances of optical components. Additionally, a tolerance analysis was performed to assess the impact of assembly uncertainties and mechanical vibrations on image quality, thereby evaluating system robustness.

Based on the optical design specifications, the acquisition of optical components started. Most parts were sourced as COTS items, except for the custom-designed VPH grism. The spectrograph’s mechanical housing was then designed to match the specified component dimensions and separation distances. Performance evaluation was carried out using key metrics, such as spectral coverage, spectral resolution, smile distortion, and keystone distortion. Finally, hyperspectral images were obtained using a pushbroom scanning technique in a rotation mode.

## 4. Spectrograph Design

### 4.1. Working Principle

**Figure 2:**
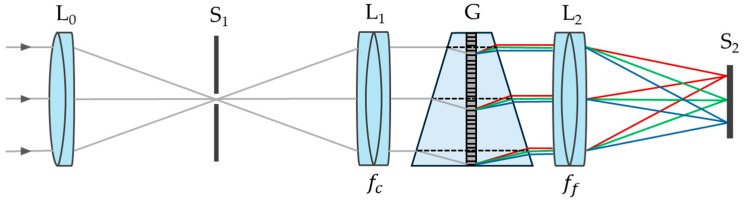
Optical diagram of HSI system, illustrating key components: a front lens (L_0_), a slit (S_1_), a collimating lens (L_1_), a VPH grism (G), a focusing lens (L_2_), and an image sensor (S_2_). Colored lines represent different wavelengths.

The spectrograph consists of several key components, as shown in Figure 2. The slit (S_1_) serves as the entrance aperture, allowing a portion of the light collected by the front lens (L_0_) to enter the system. The collimating lens (L_1_), with a focal length $f_{c}$, ensures that the light beams remain parallel before reaching the VPH grism (G), which acts as the spectral dispersion element. The dispersed light is then focused by the focusing lens (L_2_), with a focal length $f_{f}$, onto the image sensor (S_2_), where the spectral intensity corresponding to each wavelength is recorded.

**Figure 3:**
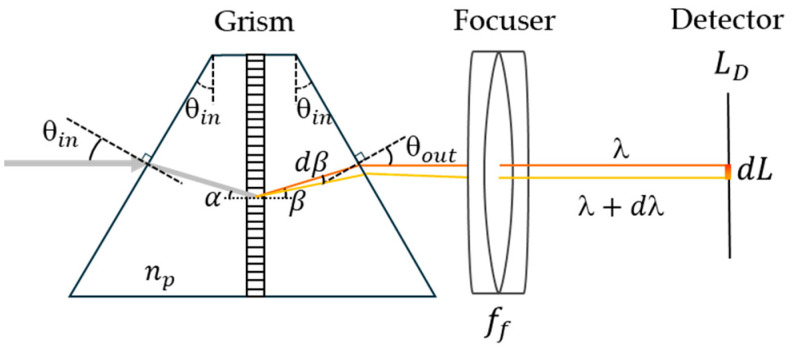
Illustration of angular dispersion of light through the grism, enabling on-axis spectrograph configuration.

A grism-enabled on-axis spectrograph configuration is illustrated in Figure 3. The collimated beam enters the first wedge prism with an incident angle of $\theta_{in}$, corresponding to the local incident angle of α at the grating interface. The diffraction angle (β) for a given wavelength is determined by the grating equation in a glass medium: $n_{p}\left(sin\alpha +sin\beta \right)=ml\lambda$, where m is the diffraction order, l is the groove density of the grating, λ is the wavelength, and $n_{p}$ is the prism refractive index. The diffracted light leaves the second wedge prism with the angle of $\theta_{out}.$ The dispersed rays produced by the grating undergo angular dispersion, which defines the correlation between the variation in diffraction angle (dβ) and the variation in wavelength (dλ). These rays are then projected onto the image sensor of length $L_{D}$ using the focusing lens. In this configuration, the primary function of the prisms in the grism is to deflect and align the global light rays along a linear path, with minimal contribution to the overall dispersion introduced into the spectrograph. This occurs because two identical prisms, oriented in opposite directions, effectively cancel out each other’s dispersion effects [28]. Consequently, the primary source of light dispersion originates from the grating. The optical analysis of the spectrograph can thus be approximated as a typical LGL system, which has a linear dispersion described by the following equation [29],

(1) $$\frac{d\lambda }{dL}=\frac{cos\beta }{ml\;f_{f}}.$$

here, dL is a spatial separation of wavelengths dλ on the detector, allowing the wavelength range $∆\lambda (\lambda_{max}-\lambda_{min})$ to be calculated using the following equation,

(2) $$∆\lambda =\left(\frac{cos\beta }{ml\;f_{f}}\right)L_{D}.$$

**Figure 4:**
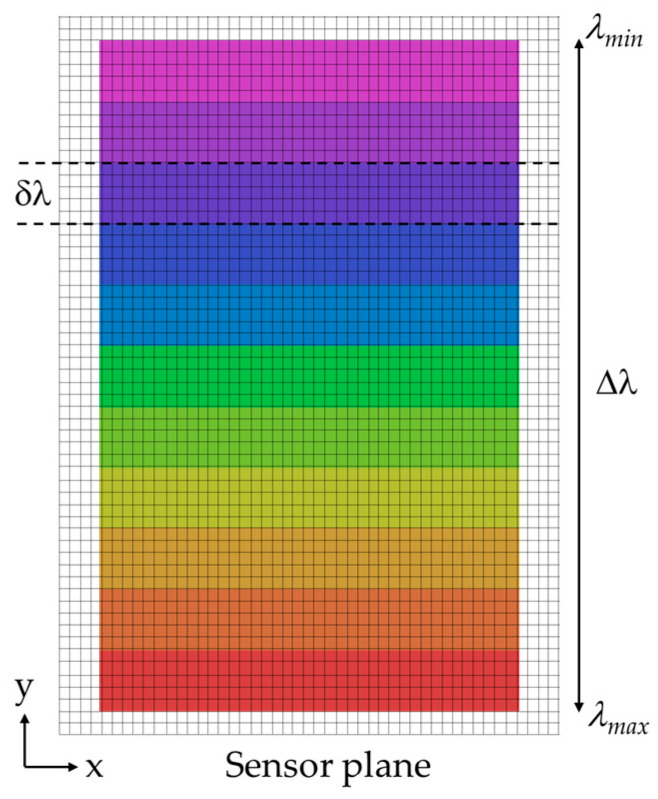
Representation of slit images corresponding to various wavelengths projected on image sensor, with spatial and spectral dimensions aligned horizontally (*x*-axis) and vertically (*y*-axis), respectively.

Additionally, the critical parameter in the design of the spectrograph is the spectral resolution (δλ), estimated by the product of the linear dispersion and the slit width [29]. For a spectrograph with unity magnification, the spectral resolution can be expressed as the following equation,

(3) $$\delta \lambda =\left(\frac{cos\beta }{ml\;f_{f}}\right)W_{s},$$

where $W_{s}$ is the slit width. The distribution of slit images corresponding to various wavelengths projected onto the image sensor is shown in Figure 4, where each grid cell represents an individual sensor pixel. In this configuration, spectral dispersion occurs along the vertical axis, resulting in spatial separation of wavelengths in the y-direction with the spectral range of ∆λ. Therefore, the number of spectral bands ($N_{b}$) that the HSI camera can resolve is estimated using the following equation,

(4) $$N_{b}=\frac{\Delta \lambda }{\delta \lambda }=\frac{L_{D}}{W_{s}}.$$

### 4.2. Specifications and Component Selection

The HSI camera in this study is designed to capture wavelengths in the VNIR regime, covering the chlorophyll-a absorption peak (680 nm), the Red Edge region (680 to 780 nm) [30,31,32], and certain atmospheric absorption features (Fraunhofer lines) with a spectral resolution finer than 5 nm [5,33]. This design makes it well-suited for remote sensing applications, including precision agriculture, environmental monitoring, and related fields. Table 2 summarizes the key specifications of the HSI system and its spectrograph components. The collimating and focusing lenses both have focal lengths of 50 mm, ensuring an image magnification close to 1. The slit measures 50 μm in width and 10 mm in length, selected to balance spectral resolution and light throughput. The spectrograph’s F-number is kept at 3 or lower to achieve a sufficient signal-to-noise ratio. A 1-inch image sensor was chosen to provide adequate spectral coverage and field of view. As indicated in Table 3, most components were sourced from COTS products supplied by Thorlabs (Newton, NJ, USA), Edmund Optics (Barrington, NJ, USA), and HuaTeng (Guangdong, China). The VPH grism was custom-manufactured by Wasatch Photonics (Logan, UT, USA).

### 4.3. Optical Simulations

**Figure 5:**
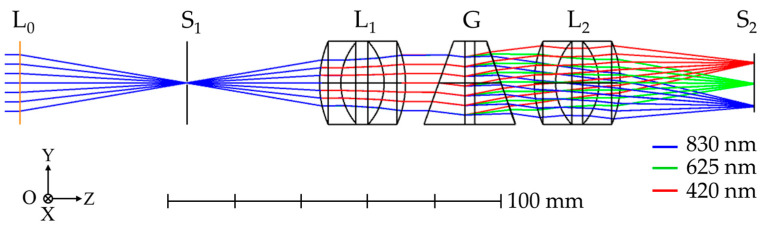
A simulation layout of grism-based spectrograph using ZEMAX OpticStudio.

Ray-tracing simulations were conducted in ZEMAX OpticStudio to determine key optical parameters of the spectrograph, including spectral coverage, spectral resolution, physical dimensions, and component spacing. The optical layout used in the simulations is shown in Figure 5 and includes a front lens (L_0_), entrance slit (S_1_), collimating lens (L_1_), VPH grism (G), focusing lens (L_2_), and image sensor (S_2_). Each element was sequentially added and aligned according to the design requirements during the construction of the optical model. For the focusing lens (L_2_), a simplified triplet lens was modeled instead of the more complex compound lens used in the actual system, which may result in slightly less accurate outcomes compared to the real system. After optimization, the spectrograph’s dimensions, component spacing, and spectral coverage were finalized. Excluding the mechanical housing and front lens, the spectrograph measures approximately 170 mm in length. Simulations indicate that the spectrograph achieves spectral coverage across the VNIR range, from approximately 420 to 830 nm.

**Figure 6:**
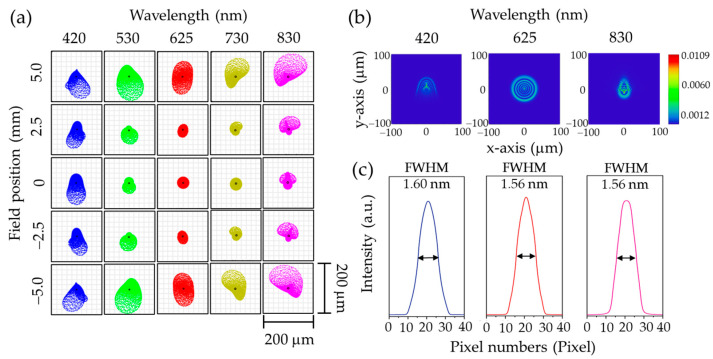
Optical simulations of grism-based spectrograph using ZEMAX OpticStudio. (**a**) A simulated spot diagram of point sources along the slit length at field positions of −5.0, −2.5, 0, 2.5, and 5.0 mm. The solid circles overlaid on each spot diagram represent the corresponding Airy disk, serving as a reference for the diffraction-limited performance of the system. (**b**) A simulation of PSF at 0 mm FOV for wavelengths of 420, 625, and 830 nm. (**c**) Estimated spectral resolution at 0 mm FOV for wavelengths of 420 nm, 625 nm, and 830 nm, obtained by calculating the FWHM of SRF(y) described in Equation (5).

The spectrograph’s optical performance was evaluated using spot diagrams at wavelengths of 420 nm, 530 nm, 625 nm, 730 nm, and 830 nm. These simulations were conducted at various field positions along the slit length within the field of view (FOV), specifically at 0 mm (the slit center, aligned with the optical axis), ±2.5 mm, and ±5 mm (corresponding to the top and bottom edges of the slit), as shown in Figure 6a. The spot diagrams indicate that spot profiles vary with field position. At the central field (0 mm), the spots are compact and symmetric, suggesting minimal optical aberrations and optimal imaging performance. However, as the field position shifts away from the center, the spots become larger and more distorted. Notably, at any given field position, the extreme wavelengths (420 nm and 830 nm) near the edges of the image sensor display more elongated and asymmetric spot profiles compared to those closer to the center, likely due to higher field angles at the focusing lens surface, which introduce increased coma and astigmatism [34]. A similar pattern appears in the point spread function (PSF) simulations at 0 mm FOV, as shown in Figure 6b. Specifically, the PSF for 625 nm, located near the center of the image sensor, is significantly more compact and symmetric than those for 420 nm and 830 nm. This observation aligns with the trends identified in the spot diagram analysis.

The spectral resolution of the spectrograph is determined by evaluating the full width at half maximum (FWHM) of its spectral response function along the dispersion direction (*y*-axis), denoted as SRF(y). This is computed by convolving several system response functions, as described by the following equation [35,36,37]:

(5) $$SRF\left(y\right)=rec\left(W_{s}\right)⊗LSF\left(y\right)⊗D\left(W_{p}\right).$$

In this equation, ⊗ denotes the convolution operator. The function $rec(W_{s})$ is a rectangular function representing the slit response, defined by the slit width $W_{s}$. LSF(y) is the line spread function along the *y*-axis, extracted from a simulation of the point spread function PSF(x,y) in ZEMAX OpticStudio, and $D(W_{p})$ is a rectangular function representing the detector response, defined by the pixel width $W_{p}$. Using $rec(W_{s})$ for a 50 µm slit width, LSF(y) extracted from the PSF simulations shown in Figure 6b, and $D(W_{p})$ for a 4.8 µm pixel width, the FWHM of SRF(y) was calculated in Python for wavelengths of 420 nm, 625 nm, and 830 nm using Equation (5), as presented in Figure 6c. The results show that the spectrograph achieves spectral resolutions of 1.60 nm, 1.56 nm, and 1.56 nm at 420 nm, 625 nm, and 830 nm, respectively.

### 4.4. Tolerance Analysis

A tolerance analysis was performed to evaluate how deviations in assembly and stability affect the spectrograph’s optical performance. This analysis was divided into two parts. The first part, termed “assembly tolerance”, examined the sensitivity of the spectrograph to misalignments caused by manufacturing errors in its mechanical housing and inaccuracies during assembly. The second part, “stability tolerance”, focused on misalignments that might occur due to vibrations and imperfections in the locking mechanisms between the optical components and the housing. The key input parameters for both analyses, as detailed in Table 4, included lens tip/tilt, lens decentering, and the distance between optical components. These parameters were systematically varied to evaluate their impact on the optical performance of the system.

**Figure 7:**
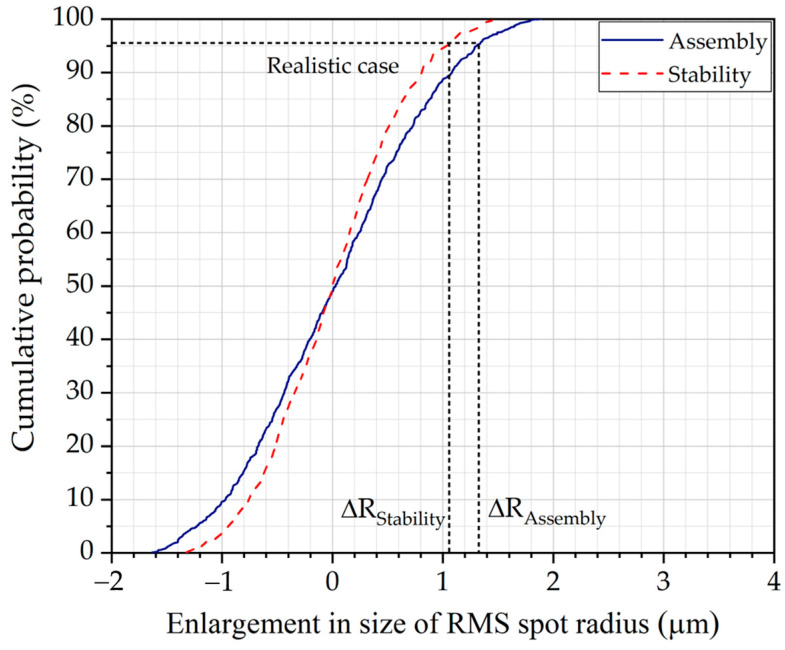
Cumulative probability curve of enlargement in size of RMS spot radius for assembly and stability tolerance analysis.

Monte Carlo simulations were performed for each tolerance analysis, generating 1000 models to assess the impact of assembly and stability errors on optical performance. For each model, the increase in the RMS spot radius at the center of the field of view was calculated at a wavelength of 633 nm. Table 5 summarizes the estimated increases in the RMS spot radius from the ideal case under typical, realistic, and worst-case scenarios represented by cumulative probabilities of 68.2%, 95.4%, and 99.7%, respectively. These values indicate the probability that the increase in RMS spot size will be less than or equal to the given values in each scenario. The overall RMS spot radius enlargement under operational conditions (${∆R}_{oc}$) was determined using the relationship $∆R_{oc}=\sqrt{{∆R}_{assembly}^{2}+∆R_{stability}^{2}}$. In addition, Figure 7 presents the cumulative probability curve of the RMS spot radius enlargement due to assembly (${∆R}_{assembly}$) and stability (${∆R}_{stability}$) errors. The spot enlargement ranges from negative to positive values, with negative values arising from the tilt of multiple components, which compensates for certain aberrations. Under realistic conditions, assembly and stability errors contribute approximately 1.3 μm and 1.1 μm to the RMS spot radius enlargement, respectively. This yields an operational-condition enlargement of 1.7 μm, indicating that assembly and stability errors have a minimal impact on the spectrograph’s optical performance. This robustness results from its on-axis configuration, which eliminates the tilting angles of optical components typically found in off-axis designs. Consequently, the grism-based HSI camera shows potential for use in high-resilience applications, such as integration with airborne vehicles and other mobile platforms.

## 5. Assembly

**Figure 8:**
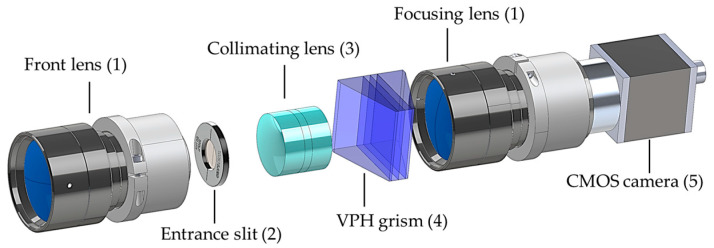
Exploded view of the HSI components, where numbers correspond to items listed in Table 3.

**Figure 9:**
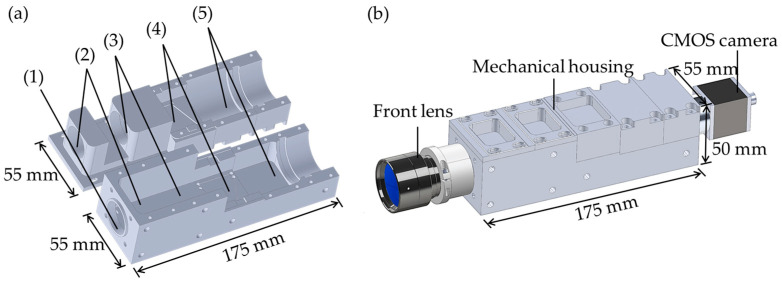
(**a**) A 3D perspective design created with SolidWorks (version 2022) for mechanical housing of HSI camera. Model comprises the following components: (1) a C-mount thread adapter for the front lens, (2) a SM1 thread adapter for a mounted slit, (3) an achromatic triplet lens holder, (4) a VPH grism holder, and (5) a focusing lens holder. (**b**) Design of final construction of HSI camera.

The on-axis configuration of the grism-based spectrograph simplifies the design and fabrication of its mechanical housing, allowing each optical component to be independently secured for precise alignment and structural stability. This approach enhances ease of assembly while maintaining optical performance. Figure 8 provides an exploded view of the spectrograph’s components, including a front lens, an entrance slit, a collimating lens, a VPH grism, a focusing lens, and a CMOS camera. Figure 9 presents a 3D model of mechanical housing designed to accommodate all optical components shown in Figure 8, created using SolidWorks (version 2022). The housing comprises two main parts: a base and covers, as shown in Figure 9a. Each optical component is precisely positioned within a machined slot, with dimensions based on manufacturer datasheets and direct measurements. The positions of these components were predetermined through ZEMAX OpticStudio simulations to ensure optimal alignment and performance. The housing base, along with the optical components, is fastened by the covers and screws. The numbered labels in Figure 9a indicate: (1) an internal C-mount thread for attaching the front lens, (2) an SM1 thread adapter for mounting the entrance slit, (3) a holder for the achromatic triplet lens, (4) a holder for the VPH grism, and (5) a holder for the focusing lens. The completed mechanical housing, excluding the front lens and CMOS camera, measures 55 mm × 50 mm × 175 mm, as depicted in Figure 9b. This compact and robust design ensures mechanical stability while preserving precise optical alignment.

**Figure 10:**
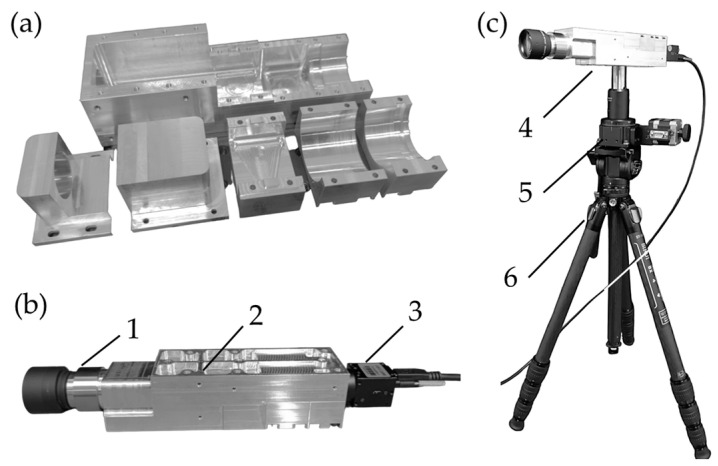
Assembled prototypes of HSI system. (**a**) Aluminum-based mechanical housing comprising a base and five covers. (**b**) Assembled housing prototype featuring (1) a front lens, (2) an HSI housing, and (3) a CMOS camera. (**c**) Pushbroom HSI prototype consisting of (4) a HSI camera, (5) a rotational motorized stage, and (6) a tripod.

The mechanical housing was fabricated using a computer numerical control (CNC) machining process. Aluminum 6061 was chosen for its lightweight properties, excellent machinability, durability, corrosion resistance, and cost-effectiveness. Figure 10a shows the aluminum-based mechanical housing, which includes a housing base and five covers, with a total weight of 701.55 g. Figure 10b presents the assembled prototype of the HSI system, consisting of (1) a front lens, (2) the HSI housing, and (3) a CMOS camera. With all optical components included, the spectrograph weighs 1.31 kg in total. Additionally, Figure 10c depicts a prototype of the pushbroom HSI system, which comprises (4) the HSI camera, (5) a rotational motorized stage (MOR-60) sourced from Optics Focus (Beijing, China), and (6) a tripod (ST-124) obtained from Sirui (Guangdong, China).

## 6. Evaluation of the Spectrograph’s Performance

### 6.1. Spectral Coverage and Spectral Resolution

**Figure 11:**
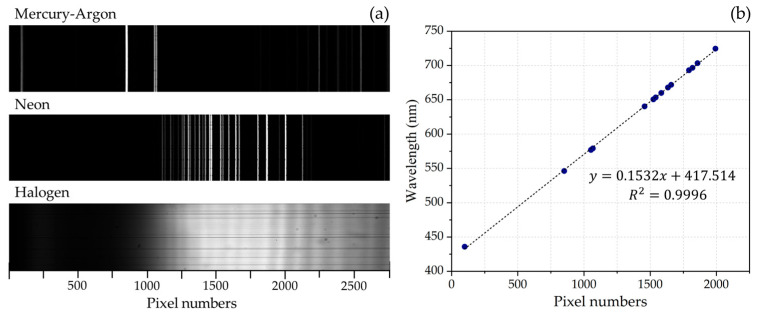
(**a**) Spectra of Hg-Ar, Ne, and halogen lamps captured by spectrograph. (**b**) Plot of wavelength versus pixel number, demonstrating a linear relationship.

**Figure 12:**
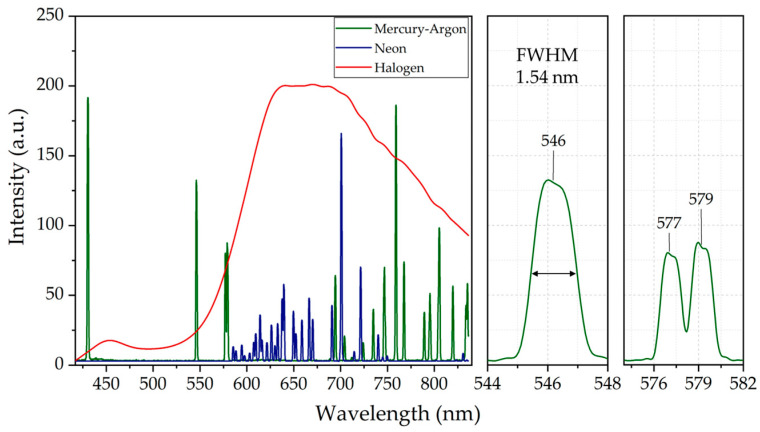
Spectra of Hg-Ar (green line), Ne (blue line), and halogen (red line) lamps measured using HSI system and displayed on a wavelength scale. System covers a wavelength range of 420–830 nm (left panel) with a spectral resolution of 1.54 nm at 546 nm (middle panel). Right panel presents two resolvable spectral peaks at 577 nm and 579 nm. Spectra were obtained by sampling the central horizontal row of the image sensor.

Following the assembly process, mercury-argon (Hg-Ar) and neon (Ne) lamps from Avantes (Apeldoorn, The Netherlands) were utilized to calibrate the spectrograph’s wavelength scale. The Hg-Ar lamp emits spectral lines at 435.8 nm, 546.1 nm, 576.1 nm, 578.2 nm, and 696.5 nm, while the Ne lamp emits lines at 667.8 nm, 671.7 nm, 692.9 nm, 703.2 nm, and 724.5 nm [38,39]. Figure 11a displays the spectra of the Hg-Ar, Ne, and halogen lamps captured by the spectrograph during calibration. Figure 11b shows the correlation between pixel numbers and peak wavelengths, revealing a strong linear relationship (R^2^ = 0.9996) and confirming high accuracy in wavelength mapping. Consequently, Figure 12 (left panel) presents spectral plots of the Hg-Ar, Ne, and halogen lamps, extracted from a central horizontal row of the image sensor, confirming a spectral range of 420 to 830 nm across the VNIR regime. The spectral resolution at 546 nm was determined by measuring the FWHM of its spectral line, as depicted in Figure 12 (middle panel), yielding a resolution of 1.54 nm. Additionally, Figure 12 (right panel) offers a magnified view of the Hg-Ar spectral peaks at 577 nm and 579 nm, demonstrating the spectrograph’s capability to resolve features with a 2 nm spectral resolution.

The FWHM analysis of dominant spectral peaks, derived from both measurements and simulations using Equation (5), is summarized in Table 6, highlighting the influence of FOV on spectral resolution. Simulation results show that the FWHM of all selected wavelengths increases at 5 mm and −5 mm FOV compared to 0 mm, closely matching the aberration patterns observed in the spot diagrams of Figure 6a. This broadening is attributed to field-dependent aberrations, primarily coma and astigmatism [34]. Minor discrepancies between measured and simulated FWHM values, particularly at 5 mm and −5 mm FOV, suggest the presence of optical distortions such as smile and keystone. These will be addressed in the following section. Despite these variations, both simulations and experimental results confirm an overall FWHM below 2 nm. Given this performance and the spectrograph’s ability to resolve closely spaced spectral peaks at 577 nm and 579 nm, the HSI camera is confirmed to achieve a 2 nm spectral resolution, enabling it to resolve 205 spectral bands across a spectral range of 420 to 830 nm.

### 6.2. Smile and Keystone

**Figure 13:**
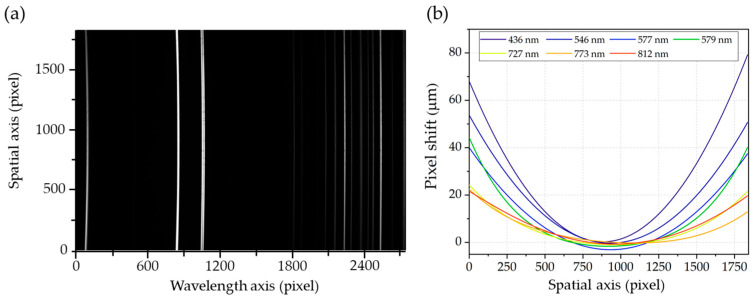
(**a**) A Hg-Ar spectrum captured by spectrograph used for measuring smile distortion. (**b**) Measured pixel shifts due to smile distortion in HSI system.

Smile and keystone distortions are significant optical aberrations that can impact the spectral and spatial fidelity of hyperspectral images [40,41]. Smile distortion, characterized by curvature in the spectral lines, leads to errors in wavelength calibration. Conversely, keystone distortion causes wavelength-dependent variations in magnification, resulting in spatial misregistration across spectral bands [42]. Consequently, precise evaluation and correction of these aberrations are critical for accurate spectral analysis and reliable image reconstruction. In this system, smile distortion was quantified by assessing the straightness of dispersed wavelengths across the FOV using an Hg-Ar light source. As shown in Figure 13a, the spectral lines display curvature indicative of smile distortion. This distortion was measured by determining the central pixel positions of each spectral line and calculating the resulting pixel shifts across the FOV. Figure 13b reveals that these shifts are most pronounced at the edges of the FOV, with an average smile distortion of approximately 28 μm (6 pixels) and a maximum of 80 μm (17 pixels) at 436 nm. Additionally, the results show that smile distortion is more prominent at shorter wavelengths and gradually decreases as wavelength increases.

**Figure 14:**
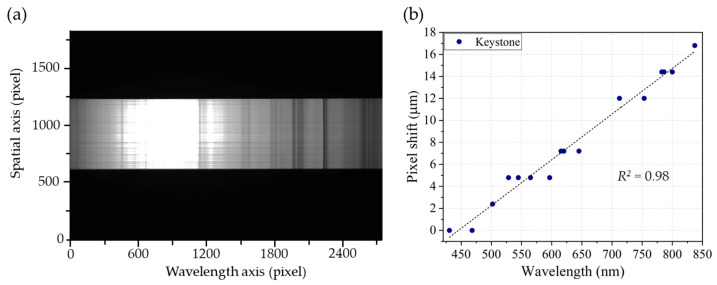
(**a**) A solar spectrum captured by spectrograph used for measuring keystone distortion. (**b**) Measured pixel shifts due to keystone distortion in HSI system.

To evaluate keystone distortion, a 3 mm slit was used in place of the 10 mm slit to ensure the entire slit image was captured by the sensor. A solar spectrum, providing a continuous range of wavelengths, served as the light source, as shown in Figure 14a. The measurement procedure was similar to that for smile distortion but was applied in the orthogonal direction. As illustrated in Figure 14b, the pixel shift due to keystone distortion increases linearly with wavelength, reaching a maximum of approximately 17 μm (four pixels) at 830 nm. These findings can be incorporated into the data processing pipeline to correct optical distortions, thereby improving data accuracy and overall system performance. Implementation of these corrective measures is planned for future work.

## 7. Acquisition of the Hyperspectral Image

**Figure 15:**
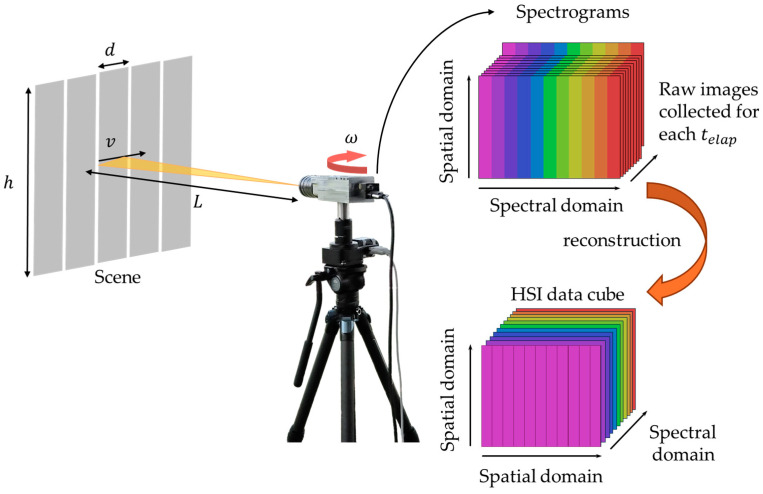
Hyperspectral data scanning using a rotational stage synchronized with camera’s frame rate. Collected spectrograms from all angular steps are reconstructed into a hyperspectral data cube.

After evaluating the optical performance of the HSI system, the next step involved demonstrating the acquisition of hyperspectral data using a rotational pushbroom scanning setup as shown in Figure 15. The in-track spatial sampling interval, d, at a scene located at a distance L away from the HSI camera is determined by the slit width $W_{s}$, and is expressed as:

(6) $$d=W_{s}\times (L/f),$$

where f is the focal length of the front lens. Similarly, the height of the scene section covered in the cross-track direction is governed by the slit height $H_{s}$, described by $h=H_{s}\times (L/f)$. To perform a rotational HSI scan, the system must ensure uniform sampling of the scene by synchronizing the camera’s frame rate (FPS) with the angular speed of the rotation (ω), while maintaining the desired spatial sampling interval. The relative linear speed of the scene swept by the camera is given by v=ωL, and the elapsed time required for the camera to sweep the sampling distance d is: $t_{elap}=d/\omega L$. To achieve continuous coverage, the angular speed of the rotational stage must be set such that the elapsed time matches the camera’s frame capture rate, i.e., $t_{elap}=FPS^{-1}$, satisfying the condition:

(7) $$\omega =\frac{W_{s}\times FPS}{f}.$$

For the camera parameters, the image capture rate (FPS) is simply the inverse of the frame capture time, $t_{cap}$, which can be tailored as, $t_{cap}=FPS^{-1}=t_{epx}+t_{read}$, where $t_{epx}$ is the camera exposure time and $t_{read}$ represents the readout time.

During the scan, the rotational stage operated at the calculated ω while the camera captured frames at the specified FPS as defined in the camera setting. This configuration allowed the slit to sweep across the scene in synchronization with the camera frame rate, ensuring sequential hyperspectral data acquisition for the sweeping scene positions. For the initial test, the bit depth of the image data was set at 8-bit and recorded in AVI format. The collected spectrograms, corresponding to each $t_{elap}$, were stored and subsequently reconstructed to generate a hyperspectral data cube. Briefly, spectrally dispersed images for each $t_{elap}$ scan were segmented into rectangular sections matching the slit dimensions, representing individual spectral band images. Images of the same spectral band were then stitched together and rearranged to construct a three-dimensional hyperspectral data cube.

**Figure 16:**
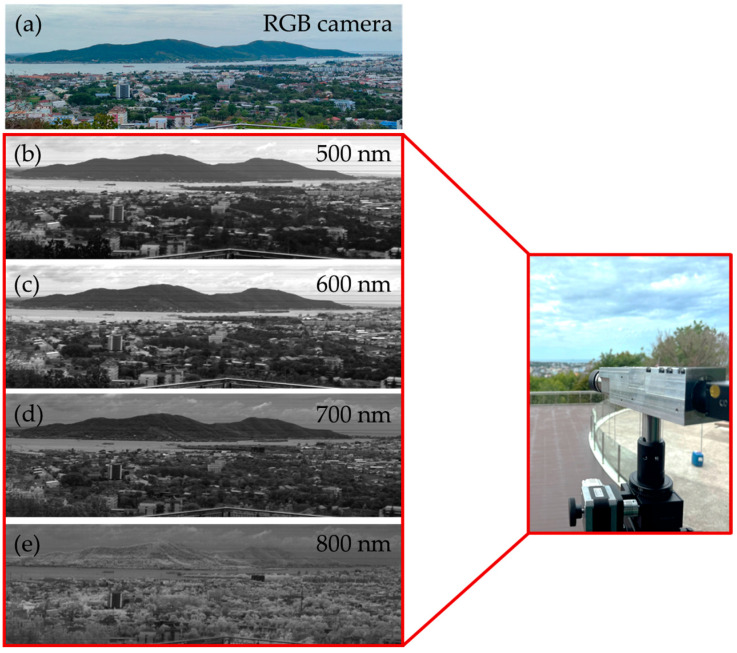
Sample images from pushbroom HSI system. Location is at regional observatory for the public, Songkhla, Thailand. (**a**) RGB camera image used as reference. (**b**–**e**) HSI images generated from hyperspectral data cube at wavelengths of (**b**) 500, (**c**) 600, (**d**) 700, and (**e**) 800 nm.

To demonstrate the capabilities of the HSI system, this prototype was used to perform a pushbroom scan at the regional public observatory in Songkhla, Thailand. Figure 16a shows an RGB image captured with a standard RGB camera, depicting the Songkhla landscape, including hills, the sea, trees, and buildings. Figure 16b–e present hyperspectral images captured by the HSI system at wavelengths of 500 nm, 600 nm, 700 nm, and 800 nm, respectively. These hyperspectral images reveal spectral characteristics of objects in the scene that are not visible in the standard RGB image. The brightness of objects in each wavelength image reflects their reflectance properties, which are tied to absorption characteristics at that specific wavelength. Brighter regions indicate higher reflectance, whereas darker regions suggest lower reflectance and thus greater absorption. A comparison of water and vegetation, as shown in Figure 16, reveals that the brightness of water decreases gradually with increasing wavelength from 500 nm to 800 nm. At 800 nm, water appears significantly darker than vegetation, indicating strong absorption at this wavelength. In contrast, vegetation exhibits high reflectance at 800 nm, appearing markedly brighter than water. This behavior aligns with the well-established optical property of vegetation, which reflects strongly in the NIR region [30]. This distinct spectral difference between water and vegetation underscores the HSI system’s ability to distinguish materials based on their absorption properties.

**Figure 17:**
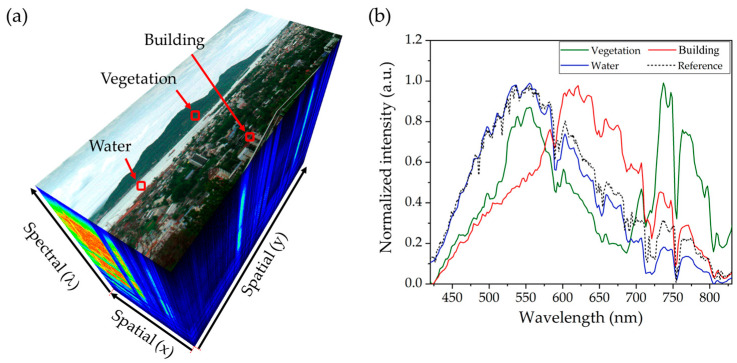
(**a**) Hyperspectral data cube captured by HSI system. (**b**) Spectral profiles of vegetation, water, and building, with solar spectrum as a reference.

To further investigate these unique characteristics, Figure 17 presents a hyperspectral data cube alongside corresponding spectral profiles of selected objects in the scene. The data cube in Figure 17a integrates spatial and spectral information, highlighting key areas such as vegetation, buildings, and water. The spectral profiles in Figure 17b depict the reflectance curves of these materials compared to the reference solar spectrum (black dotted line) measured by the HSI system. All spectral profiles reveal absorption features of Fraunhofer lines, attributed to the absorption by sodium, hydrogen, water, and oxygen in Earth’s atmosphere [43,44]. These features appear as dips in the spectral profiles at approximately 588 nm, 656 nm, 730 nm, and 760 nm, respectively. The spectral profile of vegetation (green line) exhibits distinct characteristics in the NIR region, showing strong reflectance consistent with observations in Figure 16e. It also demonstrates significant absorption in the red region around 680 nm, corresponding to the chlorophyll-a absorption band, followed by a sharp rise in reflectance between 680 nm and 780 nm, known as the Red Edge [30,31,32]. This distinctive reflectance pattern is crucial for assessing plant health and distinguishing vegetation from other objects. It also supports the development of normalized difference vegetation index (NDVI) maps and other vegetation indices, which are valuable for precision agriculture and land-use classification [45,46]. In addition, the spectral profile of water (blue line) closely matches the reference solar spectrum in the VIS region (420 to 700 nm). However, in the NIR region, it shows reduced reflectance due to strong absorption by water molecules, particularly evident between 720 nm and 730 nm [43,44]. In contrast, the spectral profile of buildings displays unique spectral characteristics, setting it apart from vegetation and water. By analyzing these spectral profiles, objects in the scene can be differentiated based on their spectral reflectance, which is linked to the optical absorption properties of their molecular composition [47]. The hyperspectral data cube thus provides a comprehensive dataset, enabling detailed analysis by leveraging the optical properties of objects within the scene. This capability is especially valuable in remote sensing applications, such as environmental monitoring, precision agriculture, and land-use classification, where accurate material differentiation is critical for effective decision-making and resource management.

**Figure 18:**
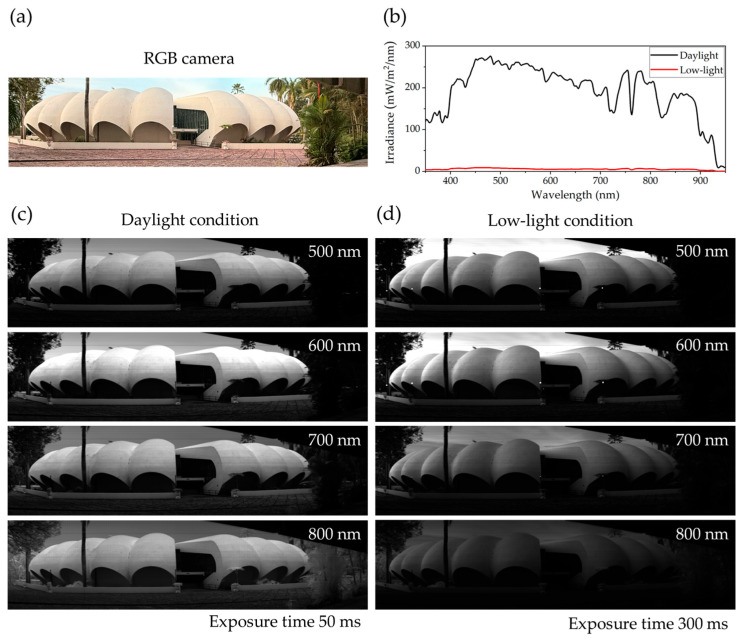
Comparison of HSI performance under different lighting conditions at Pumpkin building, Faculty of Science, Prince of Songkla University. (**a**) RGB image of scene captured by a mobile phone. (**b**) Solar irradiance measured under daylight (10:30 a.m.) and low-light (6:20 p.m.) conditions using a portable CCD Spectroradiometer (LMS-6000S). (**c**) Grayscale HSI acquired by developed HSI camera at wavelengths of 500, 600, 700, and 800 nm under daylight conditions with a 50 ms exposure time. (**d**) Corresponding images captured under low-light illumination with a 300 ms exposure time.

**Figure 19:**
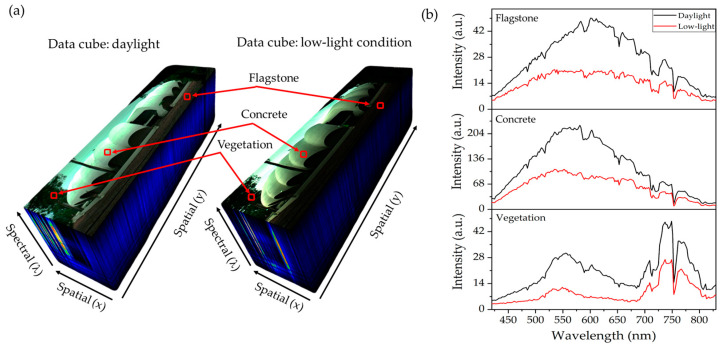
Hyperspectral data acquired under daylight and low-light conditions. (**a**) Data cubes of same scene shown in Figure 18, with selected objects including flagstone, concrete, and vegetation. (**b**) Corresponding spectral profiles showing similar patterns under low-light conditions compared to daylight, with reduced intensity but preserved distinguishable features for different objects.

Additional hyperspectral images were captured under both daylight and low-light conditions at the Pumpkin building, Faculty of Science, Prince of Songkla University, Songkhla, Thailand, to further investigate the system’s performance. A reference RGB image taken with a mobile phone is shown in Figure 18a, while Figure 18b presents the solar irradiance profiles for both lighting conditions, measured using a portable CCD Spectroradiometer (LMS-6000S, LISUN, Kowloon, Hong Kong). The irradiance measured at 6:20 p.m. under low-light conditions is significantly lower across the visible to near-infrared spectrum compared to the daylight measurement taken at 10:30 a.m. Figure 18c,d display grayscale hyperspectral images acquired by the developed HSI camera at selected wavelengths (500, 600, 700, and 800 nm). Figure 18c shows images captured under daylight with a 50 ms exposure time, while Figure 18d shows images taken under low-light conditions with a 300 ms exposure time. The daylight images in Figure 18c are consistent with those in Figure 16e, where vegetation exhibits strong reflectance at 800 nm. In contrast, the low-light images in Figure 18d show reduced detail in vegetation and other objects due to lower illumination. Despite the lower signal strength, Figure 19a,b demonstrate that the spectral signatures of vegetation, concrete, and flagstone are preserved under low-light conditions. Their spectral profiles remain similar to those under daylight, albeit with reduced intensity. Furthermore, the spectral differences among these materials are still discernible in low-light settings. These findings confirm that the developed HSI system can operate effectively in low-illumination environments without active lighting, though with some signal degradation. This demonstrates the system’s sensitivity and reliability, making it suitable for a range of scenarios, including late afternoon, overcast weather, or indoor environments with limited lighting.

## 8. Conclusions

This study introduced a comprehensive framework for developing a compact, cost-effective, and robust hyperspectral camera using COTS components and a VPH grism, advancing the accessibility of hyperspectral imaging technology. The integration of COTS components accelerated development and reduced costs, while the on-axis optical configuration, enabled by the VPH grism, optimized compactness, streamlined assembly, and bolstered system robustness, as substantiated by tolerance analysis. The resulting prototype, encased in an aluminum housing, demonstrated a spectral range of 420 to 830 nm with a 2 nm resolution across 205 spectral bands. Detailed analyses of smile and keystone distortions were conducted to guide future refinements in the data processing pipeline, further improving data accuracy. A systematic procedure for hyperspectral data acquisition via a pushbroom scanning technique was established, utilizing the HSI camera mounted on a rotating stage to capture target scenes. The collected data yielded hyperspectral images that effectively distinguished vegetation, water, and buildings, resolved Fraunhofer line absorption features, and demonstrated the HSI system’s ability to differentiate materials in low-light conditions, underscoring its potential for precise object classification. By offering a scalable, affordable, and practical solution, this work empowers researchers and engineers in remote sensing and allied fields to bridge the gap between theoretical design and real-world application. Future efforts will focus on refining distortion corrections and expanding the system’s deployment in diverse operational environments, paving the way for broader adoption and enhanced functionality in hyperspectral imaging applications.

## Figures and Tables

**Table 1:** Comparison of proposed HSI system with other on-axis HSI systems.

HSI Model	Type	Effective Specral Range (nm)	Spectral Resolution (nm)	Number of Spectral Bands
Proposed HSI Camera	Research	420–830	2	205
Imspector V8E [25]	Commercial (Specim)	350–850	5	100
Imspector V9 [26]	Commercial (Specim)	430–900	4.4	106
Imspector N10E [27]	Commercial (Specim)	400–900	2.8	178

**Table 2:** Specifications of HSI system.

Parameters	Values
Spectral coverage	VNIR
Spectral resolution	<5 nm
Number of spectral bands ($N_{b}$)	>100
F-number	≤3
Slit dimensions	50 μm × 10 mm
Focal lengths of the collimator ($f_{c}$)	50 mm
Focal lengths of the focuser ($f_{f}$)	50 mm
Pixels number (bin 2 × 2)	2744 × 1836
Pixel width ($W_{P}$) (bin 2 × 2)	4.8 μm

**Table 3:** Descriptions of components of HSI system.

Item	Part	Description	Qty
1	Edmund Optics #36-532	50 mm f/2.8, HPr Series Fixed Focal Length Lens	2
2	Thorlabs S50LK	Ø1″ Mounted Slit, 50 ± 3 µm Wide, 10 mm Long	1
3	Edmund Optics #67-422	25 × 50 mm EFL Steinheil Triplet Achromatic Lens	1
4	Wasatch Photonics	VPH Grism with 567 line/mm at 633 nm (400–1000 nm), BK7-grism, prism angle 19.6°	1
5	HuaTeng HT-SUA2000M-T	20.0 MP 1″ monochrome CMOS Sony IMX183 sensor	1

**Table 4:** Input parameters of assembly and stability tolerance analysis.

	Tolerance	Slit	Collimator	Grism	Focuser
**Assembly**	Lens tip/tilt (degree)		±0.230°	±0.460°	±0.460°
	Lens decenter (μm)		±50	±10	±100
	Distance between optical components (μm)	±50	±50	±100	±25
**Stability**	Lens tip/tilt (degree)		±0.092°	±0.092°	±0.092°
	Lens decenter (μm)		±20	±20	±20
	Distance between optical components (μm)	±20	±20	±20	±20

**Table 5:** Increased value of RMS spot radius deviated from ideal case.

Contributor	Enlargement (μm)		
	Typical Case (68.2%)	Realistic Case (95.4%)	Worse Case (99.7%)
ΔR_assembly_	0.4	1.3	1.8
ΔR_stabillity_	0.3	1.1	1.4
${∆R}_{oc}$	0.5	1.7	2.3

**Table 6:** Measurements and simulation of FWHM of dominant spectral peaks.

		FWHM					
		Measurement (nm)			Simulation (nm)		
Field Position (mm)		−5	0	5	−5	0	5
**Wavelength (nm)**	435	1.47	1.49	1.45	1.60	1.57	1.60
	546	1.54	1.54	1.50	1.70	1.56	1.70
	696	1.51	1.49	1.50	1.63	1.57	1.63
	767	1.49	1.49	1.49	1.67	1.56	1.67

## Data Availability

The original contributions presented in the study are included in the article, further inquiries can be directed to the corresponding authors.
